# Ocular fibroblast types differ in their mRNA profiles—implications for fibrosis prevention after aqueous shunt implantation

**Published:** 2013-06-12

**Authors:** Marian Löbler, Diana Buß, Christian Kastner, Jörg Mostertz, Georg Homuth, Mathias Ernst, Rudolf Guthoff, Andreas Wree, Thomas Stahnke, Georg Fuellen, Uwe Voelker, Klaus-Peter Schmitz

**Affiliations:** 1 Universität Rostock, Institut für Biomedizinische Technik, Rostock, Germany; 2 Universität Rostock, Universitätsaugenklinik Rostock, Rostock, Germany; 3 University Medicine and Ernst-Moritz-Arndt-University Greifswald, Interfaculty Institute for Genetics and Functional Genomics, Greifswald, Germany; 4 Universität Rostock, Institut für Biostatistik und Informatik in Medizin und Alternsforschung, Rostock, Germany; 5 Universität Rostock, Institut für Anatomie der Universität Rostock, Rostock, Germany

## Abstract

**Purpose:**

For an aqueous shunt draining from the anterior chamber into the choroidal space, fibroblasts from the choroidea and/or the sclera are most likely responsible for a fibrotic response around the outflow region of such a shunt. The prevention of fibrosis should extend the operating life of the shunt. A detailed characterization of fibroblasts derived from choroidea and sclera should provide information about whether a fibrosis reaction can be inhibited by cell type-specific agents.

**Methods:**

We generated mRNA profiles of fibroblasts from the choroidea, sclera, and Tenon’s space by gene array hybridization to provide a basis on which to search for potential pharmacological targets for fibrosis prevention. Hybridization data were analyzed by the Rosetta Resolver system and Limma to obtain mRNA profiles of the three fibroblast types.

**Results:**

The three fibroblast types investigated shared fibroblast-specific gene expression patterns concerning extracellular matrix proteins as collagens and fibronectin, but also showed distinct mRNA patterns.

**Conclusions:**

Individual mRNA species overexpressed in one of the fibroblast types might serve as markers for the identification of the fibroblast type in histological analyses. Future in-depth analyses of the gene expression patterns might help identify pharmacological targets for fibrosis prevention.

## Introduction

The human eye is a complex structure built from several tissue layers that function in the physiologic role of vision. Defects in one component lead to partial or total loss of vision. In glaucomatous eyes, the flow of aqueous humor from the anterior chamber is reduced, leading to an increase in intraocular pressure, which damages the visual nerve and will lead to total blindness if not treated [1]. In most cases, drugs can reduce intraocular pressure to a physiologic level [2,3]. However, for eyes resistant to the pharmacologic approach, physical drainage systems, called aqueous shunts [4], have been developed. These shunts physically drain aqueous humor from the anterior chamber to either Tenon’s space or the suprachoroidal space [5]. All devices rely on the free flow of aqueous humor into the drainage compartment. However, aqueous shunts draining into Tenon’s space induce a scarring reaction that leads to the deposition of fibrotic tissue hampering the outflow of aqueous humor [5]. Therefore, aqueous shunts draining into the suprachoroidal space between sclera and choroidea have been developed [6-8] in the hope that fibrotic reactions might be absent or negligible. Another advantage of these shunts is the existence of a physiologic suprachoroidal counterpressure to prevent severe postoperative hypotony [7]. However, fibrosis within the suprachoroidal space has been shown to be induced by an aqueous shunt draining into this space [7]. Aqueous shunts reduce intraocular pressure only for a limited period of time, because the foreign-body reaction of the eye tissues leads to fibrosis of the outflow region of the shunt. A reduction of fibrosis and preservation of aqueous humor flow over a longer period of time could partially be achieved by intra- and postoperative administration of inhibitors of fibroblast proliferation, such as mitomycin C and 5-fluorouracil [9,10].

For a focused drug administration at the outflow site of an aqueous shunt, a novel concept proposes to utilize drug delivery systems associated with the aqueous shunt to suppress fibrosis [11,12]. When the aqueous shunt drains into Tenon’s space, the fibrosis reaction is most likely caused by fibroblasts from Tenon’s space. However, when the aqueous shunt drains into the suprachoroidal space, the fibrosis is most likely caused by the fibroblasts from the choroidea and/or the sclera. To suppress fibrosis in any of these fibroblasts, it is necessary to learn whether fibroblasts from the different tissues of the eye, choroidea, and sclera are different from fibroblasts from extraocular tissue such as Tenon’s space and whether fibroblasts from the suprachoroidal space can be pharmacologically addressed to suppress fibrosis. Therefore, fibroblasts from three tissues of the eye were isolated and cultured, and gene-expression profiles at the level of RNA were generated for each fibroblast type by hybridization to DNA microarrays. Comparison of the RNA profiles shows that even fibroblasts from choroidea and sclera, which both reside in tissues defining the suprachoroidal space, differ from each other at least at the transcriptomic level, not to mention the larger differences from fibroblasts from Tenon’s space.

## Methods

### Isolation and growth of human ocular fibroblasts

Human fibroblast cell cultures from different ocular tissues were established: sclera fibroblasts (hSF), choroidea fibroblasts (hCF), and Tenon’s space fibroblasts (hTF). The use of human tissue in this study was approved by the ethics committee of the University of Rostock.

Primary cultures of human scleral and choroideal fibroblasts were prepared from donor eyeballs [13]. Briefly, the retinas were removed and the choroids separated from scleral tissues. Each tissue was cut into pieces of approximately 1 mm^2^, placed in a 12-well cell-culture plate in Dulbecco’s modified Eagle’s medium (DMEM; Applichem, Darmstadt, Germany) with 50 U/ml penicillin, 50 μg/ml streptomycin, and 10% fetal calf serum (FCS; PAA, Cölbe, Germany), and incubated at 37 °C under 5% CO_2_ at 95% humidity. Growth medium was changed three times a week. When outgrowing primary fibroblasts reached a confluent monolayer, cells were trypsinized in 0.25% trypsin/EDTA (PAA) solution in phosphate-buffered solution (PBS; 8.1 mM Na_2_HPO_4_, 1.5 mM KH_2_PO_4_, 137 mM NaCl, 2.7 mM KCl, pH 7.4) and transferred to 25 cm^2^ cell-culture flasks (Greiner Bio One, Frickenhausen, Germany).

Primary cultures of human Tenon fibroblasts were prepared after child strabismus surgery. Briefly, small pieces of nonfunctional epistler (Tenon tissues) were removed during surgery. Tissue samples were transferred into 1.5 ml caps containing DMEM without FCS, supplemented with trypsin and collagenase NB4 (Serva, Heidelberg, Germany), each at a final concentration of 2 mg/ml and incubated at 37 °C for 2 h. After tissue-digestion cells were pelleted at 250 g for 5 min, they were resuspended in DMEM containing 10% FCS and seeded into 12-well plates. After Tenon fibroblasts proliferated to a confluent monolayer, cells were trypsinized and subcultured in 25 cm^2^ cell-culture flasks.

Cells were grown to approximately 80% of confluence, split, and cultivated up to the fourth passage. At 80% confluence, cells were harvested in TRIzol^®^ (Life Technologies GmbH, Darmstadt, Germany) and used for total RNA extraction. RNA was extracted from six individual choroidea fibroblast cultures, five individual sclera fibroblast cultures, and five individual Tenon’s fibroblast cultures.

### RNA extraction

To the cells harvested in TRIzol, chloroform (Baker, Deventer, Netherlands) was added (10%), and cells were vigorously vortexed. The resulting homogenate was centrifuged at 10,000 g for 2 min, and the aqueous supernatant was reextracted with TRIzol/chloroform and centrifuged as above. The aqueous supernatant was adjusted to 35% enthanol and applied onto an RNeasy column (QIAGEN, Hilden, Germany). After washing the column, residual DNA was digested with DNase I (QIAGEN), and after three wash steps, total RNA was eluted with 100 µl of RNase free water, followed by purification using the RNA Clean-Up and Concentration Micro Kit (Norgen Biotek Corp., Thorold, ON, Canada). RNA quantity and purity was measured at 260, 280, and 320 nm with a Nanodrop photospectrometer (Thermo Scientific, Waltham, MA), and RNA integrity was assessed by using the Bioanalyzer 2100 (Agilent Technologies, Waldbronn, Germany).

### Microarray hybridization

For each sample, total RNA (200 ng) was reverse-transcribed into cDNA, subsequently amplified, and in-vitro transcribed to cRNA. Sense-strand cDNA was generated from 10 µg of purified cRNA using random primers, followed by subsequent fragmentation and labeling. Biotinylated sense-strand DNA was then hybridized to the Affymetrix GeneChip^®^ Human Gene 1.0 ST arrays (Affymetrix, High Wycombe, UK) for 16 h. The arrays were washed and stained using the Fluidics Station 450, and scanning was performed by Scanner 3000 7G (Affymetrix).

### Data preprocessing and analysis

Microarray data analysis was performed by using the Rosetta Resolver^®^ system for gene-expression data analysis (Rosetta Biosoftware, Seattle, WA). In brief, the raw signals of the probes were summarized using rubust multiarray average (RMA) [14], thereby generating probe set-specific signal intensities. Chips were normalized by using quantile normalization. Principal component analysis and agglomerative hierarchical clustering were done using the Rosetta Resolver Data Reduction and the 2D Cluster wizards (Rosetta Biosoftware). To compare RNA expression levels of genes in hCF, hSF, and hTF, normalized expression signals of genes from corresponding samples were averaged, and fold changes were calculated. To assess differences in mean signal intensities between experimental groups, analysis of variance (ANOVA, with Benjamini-Hochberg test correction) [15] and a post hoc Scheffe test were performed. Rosetta Resolver ratio-built statistics to correct for possible signal-intensity bias were also considered. Only genes with (1) an ANOVA p value of ≤0.05, (2) an absolute fold change of ≥1.5 together with a Scheffe test p value of ≤0.05 in at least one of the three pairwise comparisons (hCF vs. hTF, hSF vs. hTF, and hCF vs. hSF, respectively), and (3) a ratio-built p value of ≤0.05 were deemed differentially expressed genes (DEGs) and considered for further evaluation.

Further, hCF and hSF were merged into a metagroup, and Limma [16] was applied to identify DEGs between this metagroup of hCF and hSF and hTF alone. Limma uses a gene-wise moderated T statistic, borrowing strength from other genes, thereby increasing the confidence in the test result. A cutoff value of 0.05 for the false discovery rate and of 1.5 for the unsigned absolute fold change designate a DEG. Heat maps were constructed using pairwise Pearson’s correlation as a distance measure. Rows (genes) were scaled to a mean of zero, and a standard deviation of one (z score). A high z score represents a high mRNA abundance.

### Quantitative PCR

One µg of total RNA isolated from the fibroblasts was used for random hexamer-primed cDNA synthesis (First Strand cDNA Synthesis Kit, Fermentas, St. Leon-Rot, Germany).

The resulting cDNA was stored in aliquots equivalent to 50 ng RNA, at −20 °C. Sequences for PCR primers were generated with the Primer3 software (Whitehead Institute, Cambridge, MA) [17], and all selected sequences were compared to the available GenBank database. In this way, their identity with the target sequence was confirmed, and sequence identity or high sequence similarity to other, unrelated sequences could be excluded. Primers (Table 1) were synthesized by Eurofins MWG-Operon (Ebersberg, Germany).

**Table 1:** PCR primers.

gene symbol	accession number	left primer	right primer	amplicon (bp)
18S rRNA	NR_003286	GGTTCGAAGACGATCACATACC	TCGTTCGTTATCGGAATTAACC	344
ADAMTS12	NM_030955	CAGGGCCTGAGTCTATGAGC	TCAAGGATTGGGAAGTGAGG	186
CCRL1	NM_016557	CCTTTTTGGGCTGTTAATGC	ATGATCCAGCATGGTTTTCC	188
EMILIN2	AL117592	GCTTTAGAGGGGGAGATTGC	GCCCCTCTTTTGGATCTACC	192
GAPDH	NM_002046	CAATGACCCCTTCATTGACC	TTGATTTTGGAGGGATCTCG	159
GPR133	AL162032	ACGTCAACCTCGTGATAGGG	GGTTGGTTATGATGGGATGG	234
LRRN4CL	AL834319	AGGTGGTGACATCACAGTCG	GCATGGAGACAGTGGGTAGG	171
MGP	NM_000900	CACGAGCTCAATAGGGAAGC	CAGGGGGATACAAAATCAGG	181
OSR2	AI811298	GTGTGACATCTGCCACAAGG	CATGTGGGACATTTGTGTGG	180
RBP1	NM_002899	CAACTGGCTCCAGTCACTCC	TGCACGATCTCTTTGTCTGG	159
RGS5	AF493929	AACATCCAAGCATCGAAACC	CTCTCGTTTGCCTCAGAACC	245
SCARA5	BC033153	ACACTGAAAGTGGGCAGAGC	CCTGGTGGAAGAGAGAGACG	237
SERPINA3	NM_001085	TAAAGCCAAATGGGAGATGC	TCAGGGAGGATGAAGAGTGC	201
SLC20A1	NM_005415	GAAGAGCCGTTTGACAGAGC	TGACAGGAGGGCAGAGTACC	150

Primer sequences and amplicon size for selected genes overexpressed in hCF, hSF, or hTF.

PCR reactions were set up in quadruplicate with cDNA, primers, and PCR master mix (Thermo Scientific Finnzymes, Schwerte, Germany) containing dNTPs, polymerase, and SYBR green. After an initial denaturation step and *Thermus aquaticus* DNA polymerase activation at 98 °C for 10 min, 40 PCR cycles (94 °C 10 s denaturation, 60 °C 20 s annealing, 72 °C 30 s elongation) were run in a Master Cycler realplex^2^ (Eppendorf, Hamburg, Germany). Ct values are the means of the four parallel PCR-reactions. ΔCt and ΔΔCt values are based on these mean values. The fold-difference was calculated according to 2^ΔΔCt^ assuming an exact doubling of the PCR product with each cycle. PCR products were subjected to a melting-point analysis to check for the number of PCR products. In addition, PCR products were analyzed for size and purity on a 1.5% agarose gel.

### Histological analysis and immunohistochemistry

Human eyes were enucleated and fixed with 4% paraformaldehyde for 2 d. After fixation, ocular tissue was embedded in paraffin and 5 µm sections were mounted, dewaxed, and processed for Azan staining (azocarmine G, aniline blue, and orange G) using a standard protocol [18]. Briefly, rehydrated slides were stained in filtered and preheated (56°C) 0.1% aqueous azocarmine G (Merck, Darmstadt, Germany) solution for 15 min. After washing in distilled water slides were transferred into 0.1% aniline in 96% ethanol (Merck) until cytoplasm and connective tissue were pale pink and nuclei were well defined. Slides were rinsed with 1% acetic acid in 96% ethanol to stop further staining and to remove aniline, followed by incubation in 5% phosphotungstic acid (Merck ) for 1 h. After washing in distilled water slides were stained in 0.25% aniline blue - 1% orange G (Sigma-Aldrich, Taufkirchen, Germany; Merck) solution for 15 min and rinsed again with distilled water. Slides were dehydrated by 3 changes of absolute alcohol, cleared through 3 changes of xylene and mounted with Entellan® (Merck).

In adjacent serial sections, immunohistochemistry was performed as described elsewhere [19]. Briefly, paraffin slides were dewaxed, rehydrated, and blocked with 10% BSA in Tris-buffered saline (TBS) including 100 mM lysine and 1% Triton X100. Sections were treated for 3×5 min in a microwave (600 W) in citric acid buffer (pH 7.4), then rinsed with distilled water, followed by two washes in TBS. Then sections were incubated overnight at 4 °C with anti-collagen I antibody (Abcam, Cambridge, UK) at a 1:200 dilution. After three washes in TBS, sections were incubated in 1:1000 diluted secondary antibody (biotynilated antimouse IgG, Vector Laboratories, Burlingame, CA) for 2 h at room temperature. After three washes, sections were developed with a standard avidin-biotin-peroxidase technique (Vector Laboratories) with diaminobenzidine (Sigma, St. Louis, MO) as the chromogenic substrate, to visualize bound antibody. For control sections, the primary antibody was omitted.

## Results

### Array hybridization

**Figure 1:**
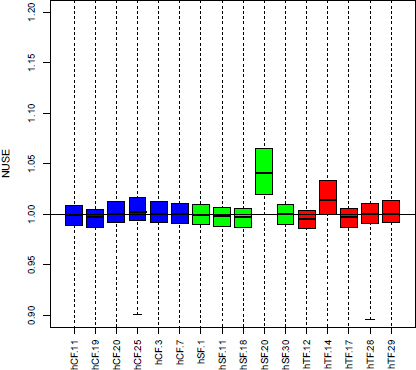
Microarray quality. This is a normalized unscaled standard error plot for assessing microarray quality. The median of one sample (hSF.20) differs considerably from value 1; this sample was therefore excluded from further analysis. The x-axis is indexed by patient ID. Choroidea fibroblasts (hCF), sclera fibroblasts (hSF), and Tenon’s space fibroblasts (hTF) designate the three cell types.

Typically 5–10 µg of total RNA were isolated from 10^6^ cells. For all samples, the RNA capillary gel electrophoresis showed two peaks in the electropherograms corresponding to the 18S and 28S rRNA and no fragmentation events. Furthermore, the RNA integrity number automatically calculated from the ratio of the major peaks and the baseline was 9.5 or higher, indicating highly intact total RNA for all samples. This was confirmed by normalized unscaled standard error plot by affyPLM (by Ben Bolstad, UC Berkeley, Berkeley, CA) [20] for assessing microarray quality. Generally, the arrays were of high quality. However, sample hSF.20 was removed (Figure 1) because its median differed considerably from that of the others, indicating either low RNA quality or problems during amplification and hybridization. In addition, the hCF.11 sample was removed to avoid doubling of patient material because cells originated from the same patient, as in sample hSF.11, bringing the final sample sizes to n=5 for hCF, n=4 for hSF, and n=5 for hTF.

### Fibroblast-specific gene expression

**Figure 2:**
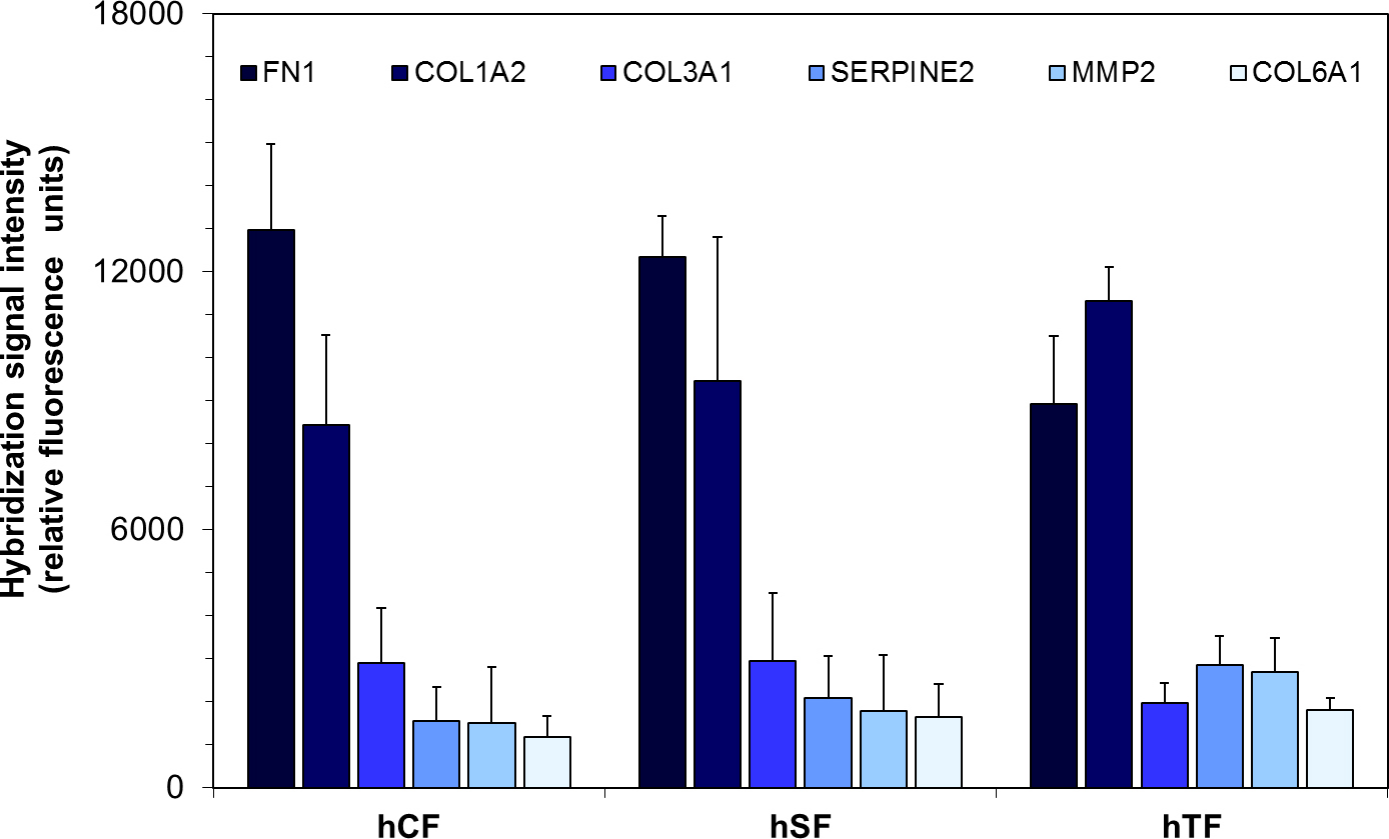
Extracellular matrix (ECM) gene expression. Comparison of the expression of distinct extracellular matrix genes in the three fibroblast populations from the choroidea (hCF), sclera (hSF), and Tenon’s space (hTF). Genes encoding fibronectin (*FN1*), collagen alpha-2(I) chain (*Col1A2*), collagen alpha-1(III) chain (*Col3A1*), glia-derived nexin (*SERPINE2*), matrix metalloproteinase-2 (*MMP2*), collagen alpha-1(VI) chain (*Col6A1*) show an expression level well above average (corresponding to a signal intensity of 134). Vertical bars indicate standard error of the mean (SEM).

Several fibroblast-specific genes, such as fibronectin (FN1), collagen types I, III, and VI (COL1A2, COL3A1, COL6A1), glia-derived nexin (SERPINE2), and matrix metalloproteinase-2 (MMP2) were found to be expressed at a level higher than average and to a comparable level in all three fibroblast types (Figure 2). Fibroblast identity could be confirmed by immunocytochemical staining with a fibroblast-specific antibody (data not shown).

### Connective tissue and collagen I expression

**Figure 3:**
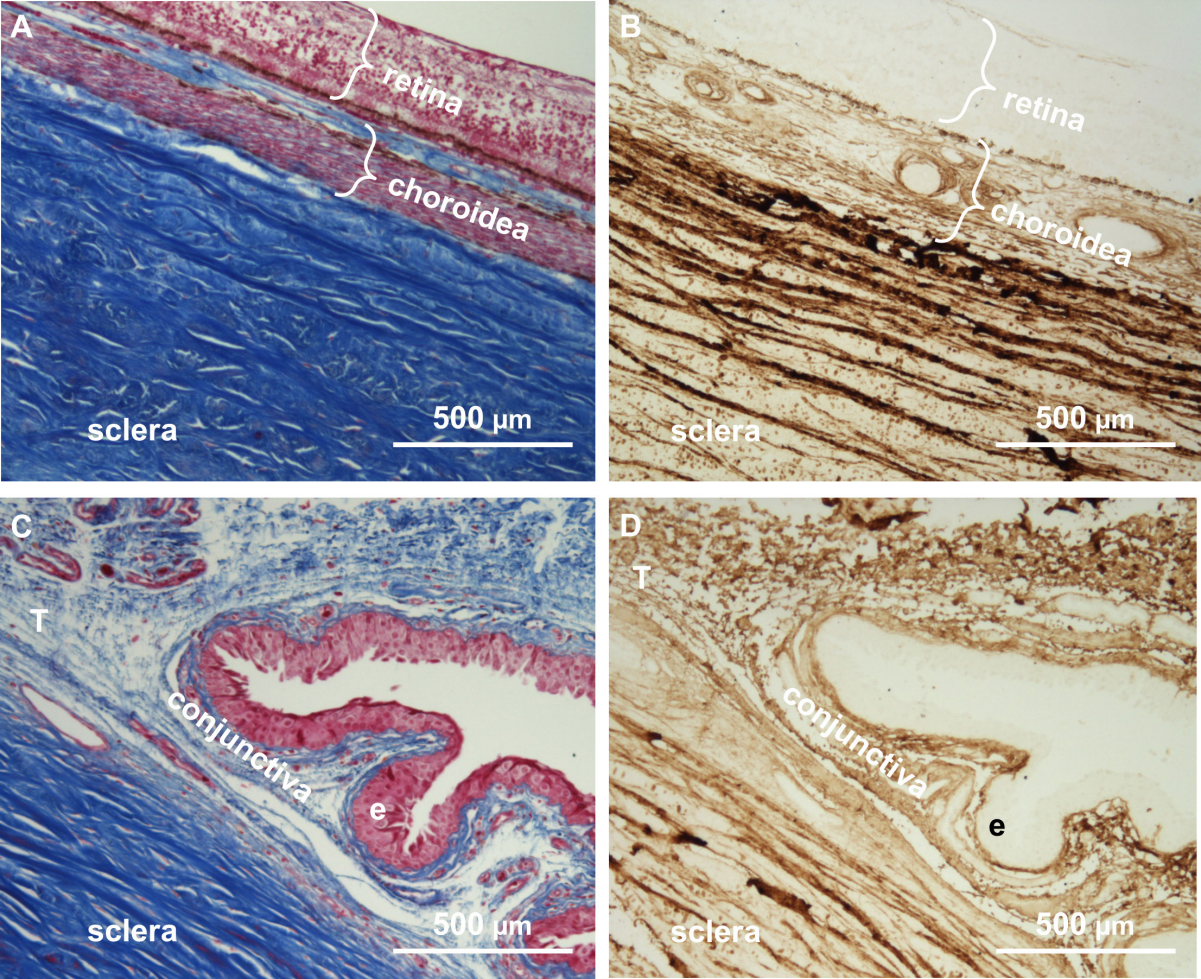
Histology and immunohistochemistry. **A**: The Cross section of the eyeball was stained for connective tissue. **B**: The Cross section of the eyeball was stained for collagen I. **C**: The cross section through the conjuntival fornix was stained for connective tissue. **D**: The cross section through the conjunctival fornix was stained for collagen I. Collagen I is absent from the retina and the conjunctival epithelium (e) but present in the small region of Tenon’s space (T), the choroid, and the sclera.

On eyeball cross sections, connective tissue was stained by the AZAN stain, and collagen I was visualized by immunohistochemistry. The blue AZAN stain was prominent in the sclera and the choroidea, indicating the presence of connective tissue, whereas the retina (Figure 3A) or the conjunctival epithelium (Figure 3C) stained red, not blue, indicating the absence of connective tissue (Figure 3A,C) The blue stain corresponded to the immunohistochemical staining of collagen I (Figure 3B,D). Again, the retina and the conjunctival epithelium layer did not contain collagen I (Figure 3B,D). Fibroblasts were obtained from three different ocular tissues: choroidea, sclera, and Tenon’s space (Figure 3).

### Distinct populations of fibroblasts

**Figure 4:**
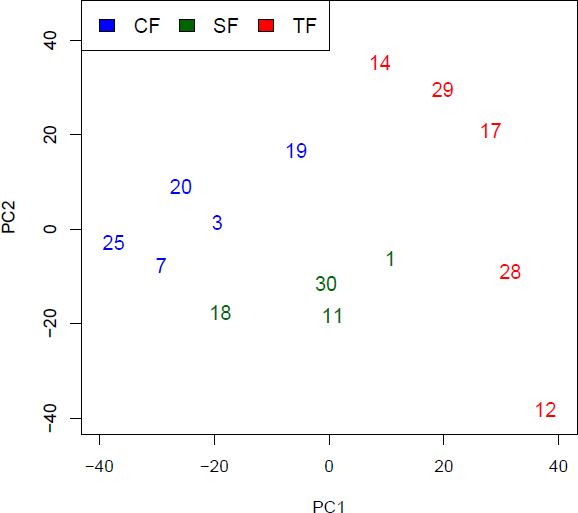
Principal component analysis: The first two principal components (PC1 and PC2) of the normalized and log-transformed gene expression data. The sample IDs were used to designate the symbols, choroidea Fibroblast (CF) sample numbers 3, 7, 19, 25 are shown in blue, sclera fibroblast sample numbers 1, 11, 18, 30 are shown in green, and Tenon fibroblast (TF) sample numbers 12, 14, 17, 28, 29 are shown in red.

Principal component analysis (PCA, Figure 4) was performed on the matrix of gene expression values. Although PCA is an unsupervised method, it clearly separates the three fibroblast types, mainly along the first principal component (PC1, x-axis in Figure 4). Furthermore, the two fibroblast types, hCF and hSF, were more similar to each other than to the Tenon’s space fibroblasts (Figure 4). The latter also appeared to have the highest intragroup variability, as judged by the high spread along the second principal component (PC2 in Figure 4).

**Figure 5:**
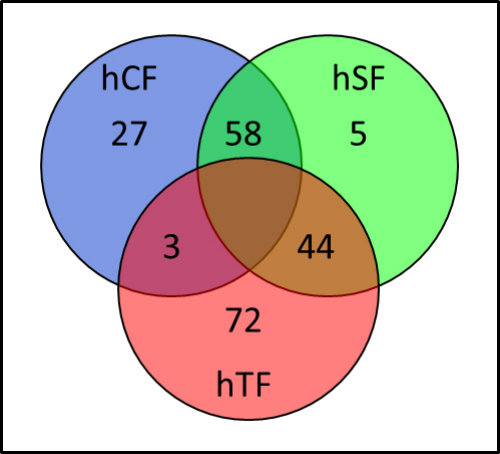
Cell type-specific mRNA abundance: The Venn diagram shows the number of genes overexpressed in each cell type in comparison to the other two. The numbers of genes overexpressed in two cell types are indicated in the intersections. The cell types are fibroblasts from the choroidea (hCF), sclera (hSF) and Tenon’s space (hTF).

Several overexpressed mRNAs could be identified for each individual fibroblast type in comparison to the other two (Figure 5). Twenty-seven mRNAs are characteristic for hCF (Appendix 1) and five for hSF (Appendix 2), whereas 58 mRNAs are overexpressed in both cell types, hCF and hSF, when compared to hTF (Appendix 4). The high number of 72 mRNAs characteristic for hTF (Appendix 3) indicated that hTFs were distinct from the other two fibroblast types hCF and hSF (Figure 5). Complete array hybridization data sets are available under GSE40929 in the Gene Expression Omnibus.

**Figure 6:**
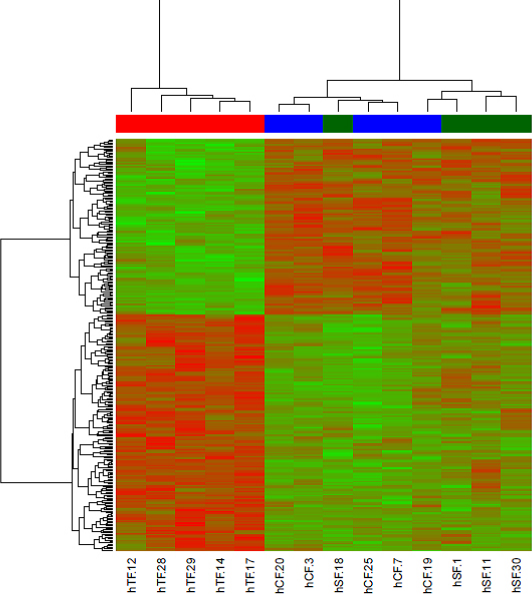
Heat map of expression values for genes (n=297) that were differentially expressed between hTF and a metagroup comprised of both hSF and hCF with a p value cutoff (Benjamin-Hochberg-corrected) of 0.05. Rows (genes) are scaled, i.e., the value (z score) for a given gene in a given sample represents its deviation from the mean expression value of the gene across all samples in terms of its standard variation, with red denoting upregulation, green downregulation. The dendrograms that determine the ordering of the rows (genes, left side) and columns (samples, upper side) are computed by hierarchical clustering using Pearson’s correlation as the distance measure and complete linkage as the clustering method.

Since the suprachoroidal space is lined by the sclera and the choroidea, scarring around an aqueous shunt draining into this space might originate from either type of fibroblast. Therefore, the gene expression pattern of these two types of fibroblasts, which are quite similar to each other (Figure 4 and Figure 5), was compared to the gene expression pattern of Tenon’s fibroblasts. A heat map of DEGs (n=297) identified by Limma [16] between hTF and a metagroup comprised of hCF and hSF (Figure 6) showed specific clustering of the samples. Although no prior knowledge regarding the membership of metagroup samples was included, hierarchical clustering based on Pearson’s correlation yielded a good separation of the fibroblast types hCF and hSF, supporting the data from principal component analysis (Figure 4) and cell type-specific mRNA abundance (Figure 5).

For each fibroblast group, at least four individual samples were analyzed by array hybridization; qPCR was performed on one additional RNA sample for each fibroblast type for differentially expressed genes. As internal reference, 18S rRNA was used, such that specific mRNA abundance was related to total cellular RNA. Also, GAPDH mRNA abundance did not differ by more than 10% between fibroblast types in array hybridization or by more than 15% in qPCR (data not shown). For qPCR, some of the overexpressed genes for each cell type identified by array hybridization were chosen randomly. Fold differences calculated from qPCR were found to be roughly comparable to fold differences calculated from array hybridization data for hCF and hTF, with the exception of the OSR2 mRNA. The latter appeared to be downregulated in hTF when compared to HSF, whereas it was upregulated when array data were considered. LRRN4CL did not show differential mRNA abundance in qPCR (Table 2) between hTF and hSF but appeared up regulated in hTF when array data are considered. No differential mRNA expression could be found in qPCR for SLC20A1 and ADAMTS12, which appeared to be overexpressed approximately two-fold in the array hybridization results.

**Table 2:** Array qPCR comparison.

**Cell type**	**Gene Symbol**	**array, fold difference**			**qPCR, fold difference**		
		versus hCF	versus hSF	versus hTF	versus hCF	versus hSF	versus hTF

**hCF**	RGS5		9.45	29.76		1.71	32.62
	MGP		5.23	11.00		38.10	25.37
	SERPINA3		4.69	5.84		10.09	23.63
	RBP1		2.77	3.07		13.18	9.60
	GAPDH		0.96	1.06		1.29	−1.15

**hSF**	SLC20A1	1.82		2.19	1.40		1.05
	ADAMTS12	2.46		3.13	−1.19		1.50
	GAPDH	1.04		1.10	−1.31		−1.27

**hTF**	SCARA5	12.00	10.33		1.67	1.89	
	OSR2	11.47	7.82		49.52	−4.69	
	GPR133	7.78	5.96		8.37	2.88	
	EMILIN2	4.43	3.95		21.30	2.69	
	LRRN4CL	4.37	4.51		2.69	−1.04	
	CCRL1	15.32	15.68		3.29	2.05	
	GAPDH	0.94	0.63		1.06	1.10	

Comparison of fold difference in mRNA abundance calculated from array hybridization (array, fold difference) and qPCR (qPCR, fold difference) between three ocular fibroblast types hCF, hSF, and hTF. The mRNA abundance of one cell type (left column) is compared to the mRNA abundance of the other two cell types. The array hybridization data are averaged over 4–5 individual samples whereas the qPCR data were obtained from one RNA sample for each cell type. The gene symbol indicates the specific mRNA. 18S rRNA served as internal reference.

## Discussion

Previously, fibroblasts that represent the outflow regions of aqueous shunts, Tenon’s space, and the suprachoroidal space were characterized on the basis of several proteins of the extracellular matrix. Some differences were observed between the three fibroblast types, but the genes that distinguish the fibroblast types were not readily usable for the goal of finding pharmacological targets for fibrosis inhibition [13]. Therefore, in this study, we conducted an extended analysis of transcriptomic data of these fibroblasts.

For such a gene expression analysis, high-quality RNA is essential to obtain solid hybridization signals. Despite good RNA quality for all samples, as determined by RNA integrity values above 9.5, in one sample, array hybridization signals suggested either partially degraded RNA or insufficient probe synthesis or hybridization (Figure 1). Even though the hybridization pattern of the “defective” RNA did not differ from parallel samples, the data of this particular hybridization were omitted from further analyses. With a few exceptions, qPCR data agreed with the array hybridization data. In cases where no differential mRNA abundance was detected by qPCR, the signal intensity of pairs of individual samples in array hybridization was found to be below a 1.5-fold difference as well. Only for OSR2 did the qPCR data clearly deviate from the array hybridization results, possibly due to a variation of mRNA abundance in hSF (Table 2).

The global gene expression analysis of the three fibroblast types revealed that fibroblasts from different compartments of the eye shared the expression of extracellular matrix (ECM) genes identifying them as fibroblasts (Figure 2). Then again, fibroblasts from different compartments of the eye did show distinct gene expression profiles (Figure 4 and Figure 5).

Due to multiple constraints, microarray experiments performed in humans often have a small sample size. This reduces the confidence in the estimation of gene-wise variances, reflected in low confidence estimates found by statistical tests that are employed for the identification of DEGs. Lee & Saeed [21] derived the sample-size estimates required to obtain a desired sensitivity and specificity. These estimates were derived under the assumption of strictly gene-wise tests for DEG identification. However, the Limma method [16] is designed to improve variability estimation for each gene by simultaneously borrowing strength from many genes by means of empirical Bayesian inferences. Therefore, Limma can make up for the small sample sizes to some extent; and indeed, it was shown to perform well when applied to spike-in experiments with sample sizes comparable to, or even smaller than, ours [22]. Naturally, our conclusions drawn from PCA are valid, irrespective of sample size.

We started with at least five tissue samples for each culture of fibroblasts originating from those tissues. All protocols were carefully designed to minimize differences due to handling conditions, and all cells were harvested after the fourth passage. The isolated RNA was analyzed for integrity, and only microarray hybridization data were included, for which the median was close to 1 (Figure 1). When different tissue samples originated from the same donor, only one was included in the study, to avoid doubling donor material.

The application of these strict criteria led to the reduction in sample sizes, yielding sample sizes of n=5 for hCF, n=4 for hSF, and n=5 for hTF. Nonetheless, the array hybridization data led to clear differences between samples by PCA (Figure 4). These differences could be confirmed for additional samples from three individual donors by qPCR (Table 2), which has strengths and limitations of its own [23]. PCA enables us to plot each individual sample, because the distances between samples reflect their similarity: short distances indicate some similarity, whereas longer distances indicate less similarity (Figure 4). Only two individual samples from the choroidea and the sclera were relatively close to each other, that is, hCF.7 and hSF.18. The similarity of fibroblast types hCF and hSF is mirrored by the low number of genes specific to one cell type when compared to the other (Figure 5). Any limitations of the array hybridization or qPCR would be identical to all samples, unless splice variants or isogenes were predominant in one or the other sample.

Removing cells from their natural ocular tissue and culturing them under artificial conditions will change gene expression patterns, which will also change over time in culture. Nonetheless, PCA still has the discriminative power to distinguish gene expression differences in the three cell types. In other words, PCA (Figure 4) is an unsupervised method, and even though gene expression data of cells from 14 individual donors were funneled through the analysis, the method separated the three fibroblast cell types (Figure 4). The high abundance of mRNAs encoding extracellular matrix proteins identified the cultured cells as fibroblasts (Figure 2). The expression of collagen I in the cultured fibroblasts can not only be explained by the cell culture on polystyrol, but is also reflected by immunohistochemistry in situ (Figure 3). Thus, despite some limitations on our approach, we have strong evidence that the differences we found were indeed due to the inherent differences between the three cell types we studied.

The similarity in gene expression profiles of hCF and hSF (Figure 4, Figure 5, and Figure 6), both bordering the suprachoroidal space, motivated us to look for specific differences, in comparison to hTF, in the gene expression of these two types, which are located outside the eyeball. The heat map (Figure 6) showed a similar pattern in the gene expression of hCF and hSF, and a clearly distinct one for hTF, corroborating the PCA. It might be argued that Tenon’s fibroblasts are obtained from young donors, whereas choroidea and sclera fibroblasts are derived from donor eyes, and that the difference in gene expression patterns might simply reflect the age difference of the original tissues. It has been shown that the proliferation potentials of young and old fibroblasts do not differ [24], whereas the duration of the cell culture changes the patterns of gene expression [25]. Therefore, great care was taken to harvest fibroblasts from the fourth passage for RNA isolation. In addition, the amount of mRNAs encoding collagen I and collagen III did not differ between fibroblasts from young and old donors [26], which was also true for the amount of collagen I and collagen III proteins [27].

From the gene expression data described here, we expect to identify targets present in hCF and hSF, which can be addressed for fibrosis prevention. When such targets can be identified, a pharmacological substance interfering with the target or an associated signaling pathway needs to be found that might serve as an antifibrotic agent in a local drug-delivery system built into the aqueous shunt [11].

One pathway common to all three fibroblast types is the TGFβ signaling pathway responsible for fibrosis [28] in cataract formation. The reduction of fibroblast proliferation by cytotoxic drugs such as paclitaxel and mitomycin C would eventually lead to reduced fibrosis, but would also be deleterious to cells in the vicinity [13]. Nevertheless, mitomycin C is effective in maintaining a low intraocular pressure after trabeculectomy [29] and in reducing fibrosis around the outflow region of an Ahmed valve, the episcleral plate [9,10]. To reduce fibrosis after aqueous shunt implantation, antiproliferative substances have been used intra- and postoperatively [9].

Our global gene expression analysis provides a wealth of data on the steady-state level of mRNA profiles of three fibroblast types of the eye. Common to all three cell types is the presence of components that belong to the TGFβ signaling pathway, which might represent the first potential pharmacological target for fibrosis prevention. In cases of cancer formation, drugs interfering with this pathway have been suggested with good results [30-36]. In terms of fibrosis inhibition, the TGFβ pathway still seems to be a valuable target [37]. Looking beyond the inhibition of the TGFβ pathway, exploring the gene expression data of the three fibroblast types to see which signaling pathways leading to fibrosis can be inhibited pharmacologically is the crucial next step.

### Conclusions

Individual mRNA species overexpressed in one of the fibroblast types might serve as markers for the identification of the fibroblast type in histological analyses. Future in-depth analyses of the gene expression patterns might help identify pharmacological targets for fibrosis prevention.

## Appendix 1. mRNAs characteristic for hCF.

Genes overexpressed in hCF in comparison to both, hSF and hTF. To access the data, click or select the words “Appendix 1.”

## Appendix 2. mRNAs characteristic for hSF.

Genes overexpressed in hSF in comparison to both, hCF and hTF. To access the data, click or select the words “Appendix 2.”

## Appendix 3. mRNAs characteristic for hTF.

Genes overexpressed in hTF in comparison to both, hCF and hSF. To access the data, click or select the words “Appendix 3.”

## Appendix 4. mRNAs of the suprachoroidal space.

Genes overexpressed in both hCF and hSF in comparison to hTF. To access the data, click or select the words “Appendix 4.”
